# Irregular adaxial–abaxial polarity rearrangement contributes to the monosymmetric-to-asymmetric transformation of *Canna indica* stamen

**DOI:** 10.1093/aobpla/plaa051

**Published:** 2020-09-11

**Authors:** Xueyi Tian, Xiaorong Li, Qianxia Yu, Haichan Zhao, Juanjuan Song, Jingping Liao

**Affiliations:** 1 Key Laboratory of Plant Resources Conservation and Sustainable Utilization, South China Botanical Garden, Chinese Academy of Sciences, Guangzhou, China; 2 National Key Laboratory of Plant Molecular Genetics, CAS Center for Excellence in Molecular Plant Sciences, Institute of Plant Physiology and Ecology, Chinese Academy of Sciences, Shanghai, China; 3 Xinxing Vocational School of Traditional Chinese Medicine, Xinxing, Guangdong, China; 4 Guangdong Provincial Key Laboratory of Applied Botany, South China Botanical Garden, Chinese Academy of Sciences, Guangzhou, China; 5 Center of Conservation Biology/Economic Botany/Plant Ecology, Core Botanical Gardens, Chinese Academy of Sciences, Guangzhou, China

**Keywords:** *Canna indica*, Zingiberales, adaxial–abaxial polarity, petaloid stamen, floral asymmetry, stamen structure

## Abstract

In flowering plants, lateral organs including stamens develop according to the precise regulation of adaxial–abaxial polarity. However, the polarity establishment process is poorly understood in asymmetric stamens. *Canna indica* (Zingiberales: Cannaceae) is a common ornamental plant with an asymmetric stamen comprising a one-theca anther and a petaloid appendage. In this study, we depicted the monosymmetric-to-asymmetric morphogenesis of *C. indica* stamen, and the morphogenesis of the monosymmetric stamen of a sister species was used as a contrast. We chose a HD-ZIP III gene family member and a YABBY family member as the adaxial and abaxial polarity marker genes, respectively, and tested their expression using mRNA *in situ* hybridization. The expression patterns of the two genes changed dynamically and asymmetrically during the stamen development process. Compared with their homologues in *Arabidopsis thaliana*, these two genes exhibited some specific expression patterns. We hypothesize that the distinctive adaxial–abaxial polarity participates in the irregular morphogenesis of *C. indica* stamen, which mediates the putative stamen-to-petaloid staminode conversion in this species.

## Teaser

In this research, we depicted the dynamic adaxial–abaxial polarity establishment process in the asymmetric stamen of Canna indica, which comprises a one-theca anther and a petaloid appendage. We used two genes from the HD-ZIP III and YABBY families as the adaxial and abaxial markers, respectively, and tested their expression with mRNA *in situ* hybridization. The polarity changed in an irregular way and is thought to be responsible for the separation of the two microsporangia within the anther and the inward curling of the petaloid appendage, thus contributing to the asymmetric development of the stamen.

## Introduction

In flowering plants, floral organs as well as other types of lateral organs are formed from the flanks of the meristems. Therefore, organ primordia have an adaxial side next to the meristem, and an abaxial one away from the meristem. When an adaxial–abaxial axis of polarity is established, it provides cues for proper structural patterning within the organ primordia (Bowman *et al.* 2002; Husbands *et al.* 2009). The morphological diversity of leaves partially relies on the differences in spatiotemporal regulation of adaxial–abaxial polarity (Fukushima and Hasebe 2014; Whitewoods *et al.* 2020). Adaxial and abaxial polarity identities are precisely regulated by two classes of genes in a mutually exclusive manner. In *Arabidopsis thaliana* (arabidopsis), *ASYMMETRIC LEAVES1* (*AS1*) and *AS2*, class III homeodomain-leucine zipper (HD-ZIP III) family genes, are involved in promoting adaxial cell fate, while *KANADIs* (*KANs*), *YABBYs* (*YABs*), *AUXIN RESPONSE FACTOR3*/*ETTIN* (*ARF3*/*ETT*) and *ARF4*, and small RNAs, such as microRNA (miRNA) miR165 and miR166, specify abaxial identity (Siegfried *et al.* 1999; Kerstetter *et al.* 2001; McConnell *et al.* 2001; Semiarti *et al.* 2001; Kidner and Martienssen 2004; Pekker *et al.* 2005; Zhong and Ye 2007; Fukushima and Hasebe 2014).

In arabidopsis, the HD-ZIP III family genes, which include *REVOLUTA* (*REV*), *PHABULOSA* (*PHB*), *PHAVOLUTA* (*PHV*), *CORONA* (*CNA*) and *ATHB8*, are repressed by miR165/6 (McConnell *et al.* 2001; Emery *et al.* 2003; Williams *et al.* 2005). The gain-of-function mutant of *PHB* exhibits adaxialized leaves. In leaves and some other lateral organs, HD-ZIP III genes function antagonistically with the abaxial identity genes, such as the KANADI family members, which positively regulate the expression of the YABBY genes (Eshed *et al.* 2001; Bowman 2004; Wu *et al.* 2007; Zhong and Ye 2007; Liu *et al.* 2011; Yang *et al.* 2014). There are six YABBY family genes in arabidopsis: *YAB1* or *FILAMENTOUS FLOWER* (*FIL*), *YAB2* to *YAB5* and *CRABS CLAW* (*CRC*), all of which exhibit a polar expression pattern and function to promote abaxial cell identity in one or more above-ground lateral organs (Sawa *et al*. 1999b; Siegfried *et al.* 1999). When YABBY genes are mutated, loss of normal polarity results in radialized leaves (Stahle *et al.* 2009; Sarojam *et al.* 2010). Besides arabidopsis, orthologous genes of *FIL* and *PHB* also show similar antagonistic expression patterns in other species such as *Antirrhinum majus* and *Cabomba caroliniana* (Golz *et al.* 2004; Yamada *et al.* 2011).

In contrast to flat organs such as leaves, the stamen development process undergoes substantial morphological changes. Correspondingly, the expression patterns of adaxial–abaxial polarity marker genes also change dynamically in this process (Sessions *et al.* 1997; Toriba *et al.* 2010; Li *et al.* 2019). In the spherical stamen primordium of rice, the adaxial marker gene is initially expressed in the domain adjacent to the meristem, and the abaxial marker is expressed on the opposite side, as in the primordia of other lateral organs. When the theca primordium emerges, a new polarity is established, with the adaxial marker expressed in the two lateral domains of the stamen primordia where the stomium would form, while the abaxial marker is expressed in the inner side of the two thecae and the connective between them. When normal adaxial–abaxial polarity is disturbed, aberrant patterning of the stamens usually occurs. In the arabidopsis *hyl1* mutant, up-regulated HD-ZIP III genes results in abnormalities in the two inner microsporangia; in *kan* and *fil* mutants, some stamens form radialized pin-like structures with all four microsporangia lost (Sawa *et al*. 1999*a*; Eshed *et al.* 2001; Lian *et al.* 2013). In general, stamens are monosymmetric with the adaxial–abaxial axis as the axis of symmetry. However, asymmetrically patterned stamens exist in some mutants (Toriba *et al.* 2010; Li *et al.* 2019). In the *rod-like lemma* (*rol*) mutant of rice, three types of aberrant stamen develop. In the case of an intermediate defect, only half of the stamen primordia establishes adaxial–abaxial polarity to form one theca, which leads to the asymmetric pattern of the stamens (Toriba *et al.* 2010). Besides, natural asymmetric stamen can also be found in some species, such as *Canna indica*, a Zingiberales ornamental plant.

The Zingiberales comprises eight families, including Musaceae, Strelitziaceae, Heliconiaceae, Lowiaceae, Zingiberaceae, Costaceae, Marantaceae and Cannaceae (Tomlinson 1962; Dahlgren and Rasmussen 1983; Kress 1990; APG IV 2016). Many species in the Zingiberales are economic plants which are widely cultivated, including banana, ginger and *C. indica* (Bartlett and Specht 2010; Tian *et al.* 2016). In this order, not all the androecial members develop into fertile stamens due to the tapeloid, petaloid homeotic transformations or total abortion of these organs. In banana families (Musaceae, Strelitziaceae, Lowiaceae and Heliconiaceae), the number of fertile stamens is five or six, while in the other four families (the ginger families), the number reduces to one in the Costaceae and Zingiberaceae and one-half in the Marantaceae and Cannaceae. Flowers of *C. indica* (Cannaceae) comprise 3–4 (occasionally five) petaloid staminodes and a half-petaloid stamen. The stamen consists of a one-theca anther and an associated petaloid appendage and exhibits an asymmetric pattern (Kirchoff 1983; Rudall and Bateman 2004; Miao *et al.* 2014; Tian *et al.* 2018).

In our previous research, ectopic expression of *PHB* was shown to inhibit the development of microsporangium in the stamen of arabidopsis, whereas *PHB* normally promotes the formation of the internal boundary between inner and outer microsporangia within the anthers (Li *et al.* 2019). We have undertaken a preliminary analysis of the adaxial–abaxial polarity in the asymmetric stamen of *C. indica* (Tian *et al.* 2018), but the detailed structural patterning process of the asymmetric stamen which, significantly, controls the symmetry of the whole flower, is not well understood. In this study, we examined the adaxial–abaxial polarity throughout the stamen development process of *C. indica*, and used the monosymmetric stamens of *Alpinia galanga* (Zingiberaceae) and arabidopsis as necessary contrasts. The marker genes exhibited dynamic asymmetric expression patterns during the asymmetric development process of *C. indica* stamen. These results provide fundamental data for understanding the irregular stamen structural patterning of *C. indica*.

## Methods

### Plant materials

For morphological studies, *C. indica* and *A. galanga* plants were grown in South China Botanical Garden (Guangzhou, China). For *in situ* hybridization, *C. indica* plants were grown in a growth chamber in the phytotron (22 °C with 16 h of light per day). The seeds of arabidopsis Landsberg erecta (Ler) were surface sterilized in 70 % (v/v) ethanol for 1 min and then in 1 % (v/v) NaOCl for 10 min. Then they were washed four times with sterile distilled water. The seeds were then placed on the surface of 1 % (w/v) agar-solidified Murashige and Skoog medium. Plates were sealed with Parafilm, incubated at 4 °C in darkness for 3 days and moved to the growth chamber at 22 °C with a 16-h photoperiod.

### Scanning electron microscopy (SEM)

Young inflorescences of *C. indica* and *A. galanga* with bracts and/or large sepals and petals removed were fixed in FAA (90 parts 70 % ethyl alcohol: 5 parts glacial acetic acid: 5 parts 40 % formaldehyde). The samples were dehydrated in an alcohol series (75, 85, 95, 100 and 100 %) before transfer to isoamyl acetate. The samples were then critical point dried with CO_2_, mounted on stubs, gold-coated in the JFC-1600 Auto Fine Coater (JEOL, Tokyo, Japan) and observed under a JSM-6360LV SEM (JEOL).

### Light microscopy

*C. indica* flowers were fixed in FAA for 48 h, and then dehydrated for successive 30 min periods in 80, 90, 100 and 100 % ethanol before being embedded in paraffin. Transverse serial sections (7 μm) were cut using a microtome and mounted on slides. These sections were stained with Safranine T (50 % ethanol) and fast green (95 % ethanol). A Zeiss Axiophoto microscope equipped with a 5-megapixel QImaging digital camera was used to take photomicrographs.

### *In situ* hybridization

Seven-micron thick sections of young inflorescences of *C. indica* (with the phyllary removed) and arabidopsis were prepared according to pre-treatment and hybridization methods described previously (Brewer *et al.* 2006). Digoxigenin-labelled hybridization probes of *CiPHB1*, *CiFIL*, *AtPHB* and *AtFIL* were prepared by *in vitro* transcription following the manufacturer’s protocol. Gene information and primer pairs used are listed in **Supporting Information—Table S1**.

## Results

### Indirect stamen initiation from the meristem

**Figure 1.**
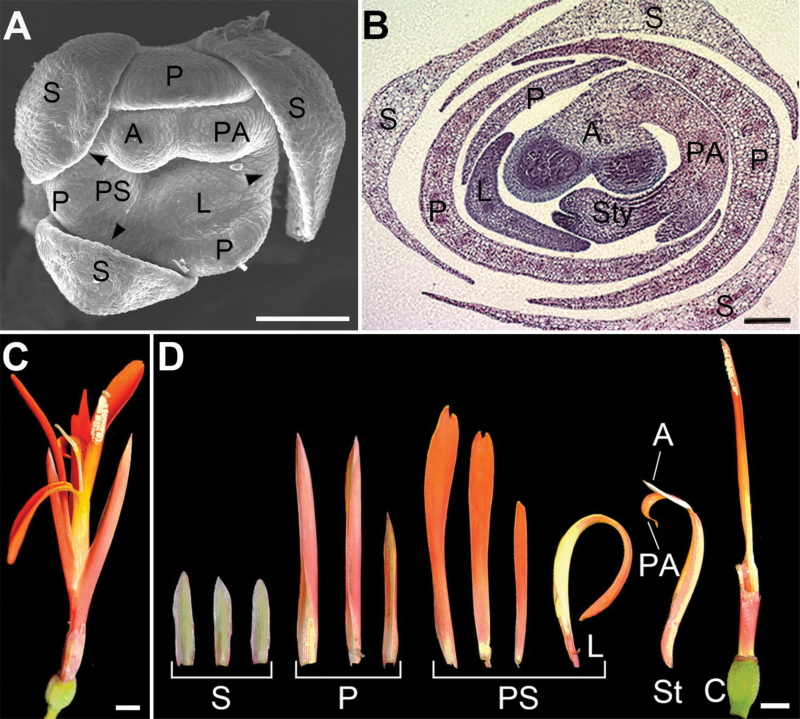
Flower traits of *C. indica*. (A) A young flower at floral organ initiation stage. Arrowheads indicate the organs of outer androecial whorl. (B) Transverse section of an older flower. (C) A flower at anthesis. (D) Dissected floral organs of a mature flower. S: sepal; P: petal; PS: petaloid staminode; L: labellum; St: stamen; A: anther; PA: petaloid appendage; C: carpel; Sty: style. Bars = 100 μm in (A and B) and 5 mm in (C and D).

**Figure 2.**
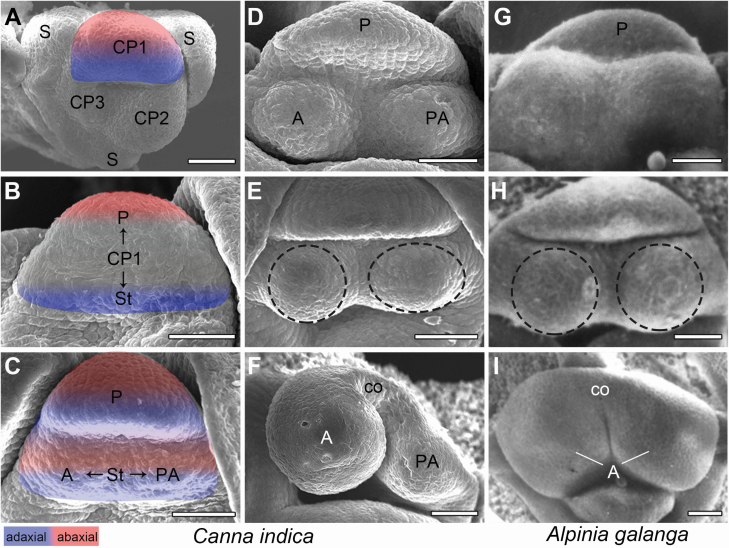
Stamen development processes of *C. indica* and *A. galanga.* (A–C) Stamen initiation process and the corresponding adaxial–abaxial pattern changes of *C. indica*. (D–F) Asymmetric stamen development process of *C. indica*. (G–I) Monosymmetric stamen development process of *A. galanga.* The dashed circles indicate the preliminary morphological difference between anther and petaloid appendage in *C. indica* (B) and the same round shapes of the two growth centres in *A. galanga* (H). S: sepal; CP: common primordium; P: petal; St: stamen; A: anther; PA: petaloid appendage; co: connective. Bars = 50 μm.

In the flowers of *C. indica*, due to the differentiation of the androecium to fertile stamen and petaloid staminodes, there are five kinds of floral organs: sepals, petals, petaloid staminodes, stamen and carpels. Flat organs are the dominant type, including three sepals, three petals and 3–5 petaloid staminodes (Fig. 1). In the flower primordium, sepal primordia are initiated first, followed by three common primordia arranged in a ring (Fig. 2A). Androecium members and petals are initiated from the common primordia, rather than from the meristem directly. Initially, the first common primordia has an adaxial side and an abaxial side. When it has differentiated into a petal primordium and a stamen primordium, each of these two organ possesses an adaxial side and an abaxial side (Fig. 2B and C).

### Asymmetric development process of *C. indica* stamen

The fertile stamen of *C. indica* comprises an anther and a petaloid appendage. During the differentiation process, these two structures experience significant morphological changes. In order to get a clear and comprehensive understanding of the pattern change process, we compared the differentiation process of the *C. indica* stamen with the two-thecae stamen of *A. galanga* (Fig. 2D–I).

The stamen of *A. galanga* derives from a petal-stamen common primordium, just like that in *C. indica*. In each species, when the stamen primordium has separated from the petal primordium, there is a pair of symmetric growth centres within it (Fig. 2D and G). Later, the left-hand growth centre (anther primordium) of *C. indica* as well as the two stamen growth centres of *A. galanga* keeps a round shape, while the right-hand one (petaloid appendage primordium) is slightly laterally elongated, forming an oval-shaped structure (Fig. 2E and H). As the primordial connective region forms on the abaxial side of the stamen primordium in *C. indica*, the anther primordium grows apparently larger than the petaloid appendage primordium. The petaloid appendage gradually turns flat, and becomes more and more different from the spherical anther (Fig. 2F). Thus, an asymmetric pattern is established in the *C. indica* stamen. At later developmental stages before anthesis, the margin of the petaloid appendage curves clockwise and constrains the style tightly to the pollen-releasing anther (Fig. 1B), thus facilitating the secondary pollen presentation on the flat style (Maasvan and Maas 2008). On the other hand, the two parts of the *A. galanga* stamen remain identical to each other, and eventually give rise to a monosymmetric two-thecae stamen (Fig. 2I).

In summary, in the two species, the stamens are both monosymmetric in the beginning, but end up as different structures with different symmetries. However, the anther primordium of *C. indica* develops in a similar manner to the left half of the *A. galanga* stamen, and the petaloid appendage experiences unique structural changes on the right-hand side of the stamen.

### Antagonistic expression of *CiPHB1* and *CiFIL* in floral organs

We used *CiPHB1* and *CiFIL* as the marker genes for adaxial and abaxial polarities respectively (Tian *et al.* 2018), and examined their expression patterns in developing flowers using *in situ* hybridization.

**Figure 3.**
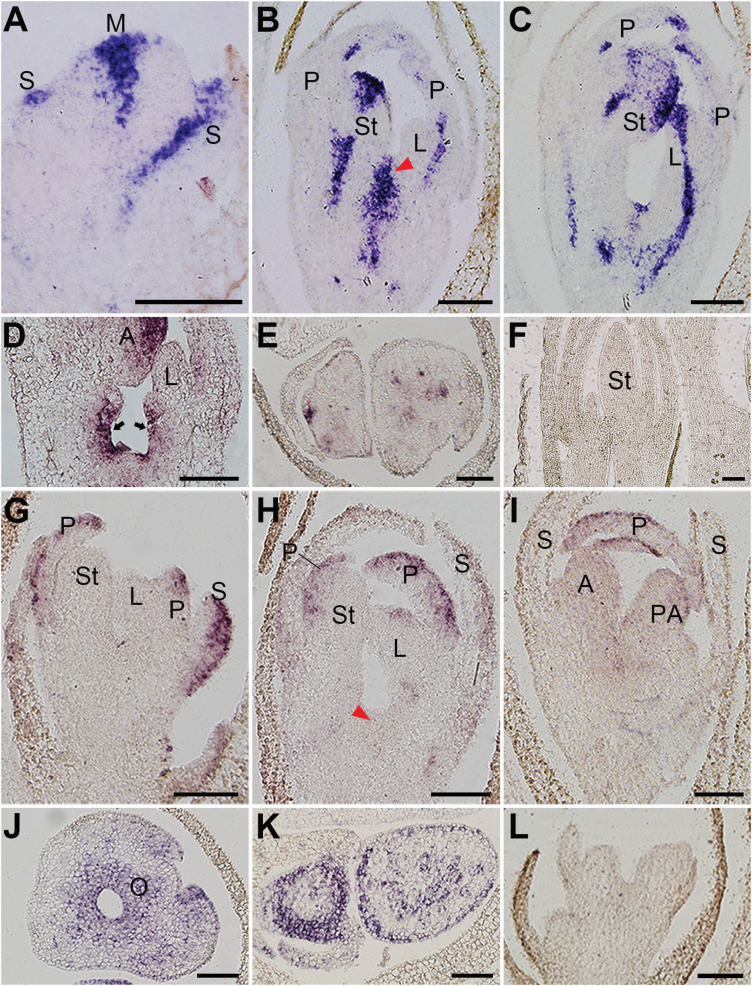
Expressions of *CiPHB1* and *CiFIL* during flower development. (A–E) Expressions of *CiPHB1* in petals, stamen, labellum (A–C), ovary (D) and pedicel (E). (G–K) Expressions of *CiFIL* in sepals, petals, stamen, labellum (G–I), ovary (J) and pedicel (K). (F and L) Sections with sense probes of *CiPHB1* (F) and *CiFIL* (L) as negative controls. Arrows in (D) indicate the expression of *CiPHB1* in the ovary. Red arrowheads in (B and H) indicate the central floral region where carpel primordia would be initiated later. Sections in (E,J,K) are transverse and all the others are longitudinal. M: meristem; S: sepal; P: petal; St: stamen; L: labellum; A: anther; PA: petaloid appendage; O: ovary. Bars = 100 μm.

*CiPHB1* was expressed in the centre of the floral meristem and on the adaxial side of the sepal primordia (Fig. 3A). Later, *CiPHB1* transcripts were detected in different organs including petals, petaloid staminodes, stamen, carpels, and pedicels (Fig. 3B–E; Tian *et al.* 2018). Expression was also observed in the developing vascular bundles (Fig. 3B,C,E; McConnell *et al.* 2001). While *CiFIL* was expressed in the abaxial sides of the sepals, petals and petaloid staminode primordia (Fig. 3G–I), *CiFIL* transcripts were also detected in the ovary and the abaxial surface of the pedicels (Fig. 3J and K).

Compared with *CiFIL*, the polarized expression patterns of *CiPHB1* were less evident and persistent (Fig. 3B and H). However, they showed antagonistic expression in several domains. In sepal primordia, *CiPHB1* was expressed in the adaxial domains, while *CiFIL* was in the abaxial domains (Fig. 3A,G,H); *CiPHB1* signals were detected in the central region of the young flower where the carpel primordia would emerge later, while *CiFIL* signals were not (Fig. 3B and H). These results verified the applicability of the two genes as adaxial and abaxial markers, respectively.

### Dynamic expression patterns of *CiPHB1* and *CiFIL* during stamen morphogenesis

**Figure 4.**
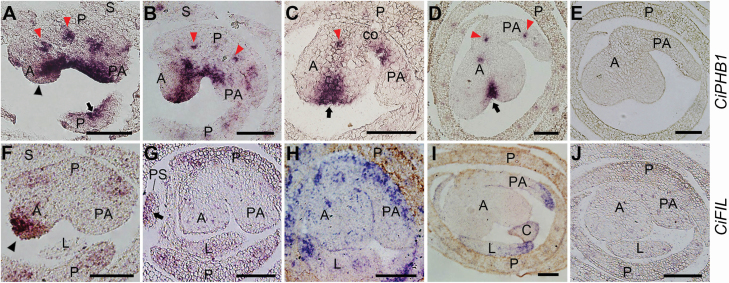
Temporal-spatial expression of adaxial and abaxial marker genes in the stamens of *C. indica*. (A–D) Expressions of *CiPHB1* during stamen development process in *C. indica*. (F–I) Expressions of *CiFIL* during stamen development process in *C. indica*. (E and J) Sections with sense probes of *CiPHB1* (E) and *CiFIL* (J) as negative controls. Arrows in (A) and (G) indicate the adaxial and abaxial expression of *CiPHB1* and *CiFIL* in floral organs respectively. Arrows in (C and D) indicate the *CiPHB1* expression in the stomium region between the two anther locules (microsporangium). Black arrowheads in (A) and (F) indicate the fore-end of anther primordium. Red arrowheads in (A–D) indicate the expression of *CiPHB1* in vascular bundles. All the sections are transverse. A: anther; PA: petaloid appendage; S: sepal; P: petal; PS: petaloid staminode; L: labellum; C: carpel; co: connective. Bars = 100 μm.

After the regular organs, we examined the expression patterns of *CiPHB1* and *CiFIL* in the irregular shaped stamen. Before the separation of petal and stamen was completed, *CiPHB1* was expressed in the adaxial side of the stamen primordium, with the exception of the fore-end of the anther region (Fig. 4A). Meanwhile, *CiFIL* transcripts were detected at the fore-end of the anther primordium but not in the other parts, exhibiting a partially antagonistic expression pattern with *CiPHB1* (Fig. 4F). After that, the *CiPHB1* expression domain extended to the front edge of the anther primordium, separating the thecae primordium into two parts, and shrank in the petaloid appendage primordium (Fig. 4B). At the same stage, *CiFIL* transcripts disappeared from the anther primordium (Fig. 4G). When the stamen primordium had completely separated with the petal primordium and obtained a free abaxial side, *CiFIL* expression was observed at this region (Fig. 4H). Meanwhile, *CiPHB1* transcripts were found on the adaxial side of the connective region. In addition, as the anther primordium grew larger, the main expression domain of *CiPHB1* was confined in the middle of the anther fore-end, where the stomium would eventually form (Fig. 4C). As the signal of *CiPHB1* faded in the connective region (Fig. 4D), *CiFIL* transcripts lost their abaxial preference, and started to accumulate in the petaloid appendage primordium slightly (Tian *et al.* 2018). Eventually, *CiFIL* expression contracted to the margin of the petaloid appendage (Fig. 4I). When the expression of *CiPHB1* declined in the adaxial domains of the lateral organs, the transcripts were continuously detected in the vasculature (Fig. 4A–D).

### Expression patterns of *AtPHB* and *AtFIL* in the stamen of arabidopsis

**Figure 5.**
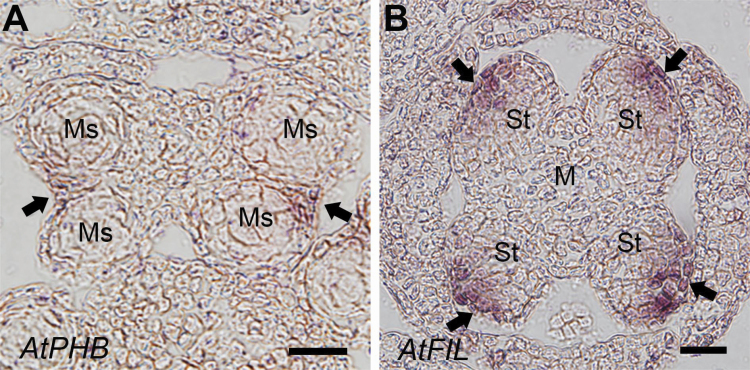
Expressions of adaxial and abaxial marker genes in the stamens of arabidopsis. (A) Expression of *AtPHB* in the anther of arabidopsis. (B) Expression of *AtFIL* at stamen initiation stage in the flower of arabidopsis. The expression domains are indicated with arrows. Ms: microsporangium; M: meristem; St: stamen. Bars = 20 μm.

The expression patterns of the adaxial and abaxial polarity genes in the stamen of arabidopsis were also tested as contrasts. *AtPHB* and *AtFIL*, which are the homologues of *CiPHB1* and *CiFIL*, were used as marker genes. As the expression patterns of these two genes have been reported before (Siegfried *et al.* 1999; Li *et al.* 2019), only some typical data is shown here. In the young stamen of arabidopsis, when the four-microsporangia pattern was established, *AtPHB* expression in the anther was restricted in the stomium regions between the inner and outer microsporangia (Fig. 5A). Apart from there being two symmetric thecae, the location of *AtPHB* expression in the stomium region was similar to that in *C. indica* (Fig. 4D). However, at an earlier stage, when the four medial (long) stamens were emerging from the central meristem, *AtFIL* was expressed in the abaxial domains of the stamen primordia (Fig. 5B), in contrast to the expression pattern observed for *CiFIL* in *C. indica*.

## Discussion

Petaloidy of androecial organs is a representative character in the flowers of *C. indica* as well as many other Zingiberales species, and it is also an important clue for analysing the evolution of this order (Kress *et al.* 2001; Wake *et al.* 2011; Specht *et al.* 2012; Fu *et al.* 2014). Compared with the two-thecae stamen in the Zingiberaceae, half of the stamen is replaced by a petaloid appendage in the Cannaceae (Kirchoff 1991). In addition, irregular petaloidy in the androecium results in the asymmetric one-theca stamen, and provides good material for studying organ identity specification and floral structure patterning in the Zingiberales.

### Distinctive expression patterns of *CiPHB1* and *CiFIL* in *C. indica* stamen

**Figure 6.**
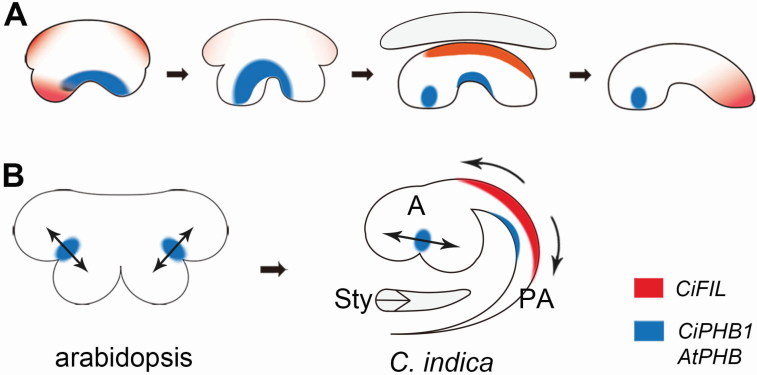
Models for structural patterning in *C. indica* stamen. (A) Summary for adaxial–abaxial polarity establishment during stamen development of *C. indica*. (B) Stamen structural patterning of arabidopsis and *C. indica*. Double headed arrows indicate the separation of two microsporangia by *AtPHB* or *CiPHB1* expression. The expression of *CiPHB1* and *CiFIL* in the middle region of the petaloid appendage possibly promote the inward curling of this structure, which enwraps the style at later stages. The gene expression is earlier than the structural curling, yet they are combined in one presentation for simplicity. A: anther; PA: petaloid appendage; Sty: style.

In the stamens of rice and arabidopsis, the expression patterns of adaxial–abaxial polarity marker genes change dynamically throughout the development process (Sessions *et al.* 1997; Toriba *et al.* 2010; Li *et al.* 2019). Similarly, *CiPHB1* and *CiFIL* also exhibited dynamic expression patterns in the stamen of *C. indica* (Fig. 6A). However, as there is only one theca in the *C. indica* stamen, the exact change process is clearly different from that in rice and arabidopsis. Besides, there are species-specific features that make the change process distinctive and rather complicated.

In the stamens of rice and arabidopsis, the adaxial–abaxial polarity is initially established as in other lateral organs, which show monosymmetric patterns (Sessions *et al.* 1997; Toriba *et al.* 2010; Li *et al.* 2019). Whereas, in the stamen of *C. indica* asymmetric expression patterns occur. As the stamen primordium differentiated from the petal primordium, *CiFIL* showed expression at the fore-end of the anther primordium (Figs 4F and 6A). Although *CiPHB1* was antagonistically expressed on the adaxial side of the remainder of the stamen primordium, it is not appropriate to regard the expression of *CiFIL* here as abaxial-type. One reason for this is that the expression domain of *CiFIL* here was on the adaxial side of the anther, and another is that a similar expression domain was not symmetrically observed in the petaloid appendage. Perhaps besides specifying abaxial identity, *CiFIL* is involved in the initiation of sporogenesis. However, the exact function of *CiFIL* here is not known, and more detailed research is needed.

In arabidopsis, *AtFIL* was expressed in the abaxial domains of stamen primordia at the initiation stage (Fig. 5B; Siegfried *et al.* 1999). While in *C. indica*, as the stamen originates from a common primordium rather than from the central meristem directly, *CiFIL* did not show abaxial expression in the stamen until it separated with the petal and obtained a free abaxial side for itself (Figs 4H and 6A). This kind of expression pattern contributes to the specificity of stamen polarity in *C. indica*. At later stages, the adaxial marker was mainly expressed in the region between the two protrusions of the theca, while the abaxial marker was expressed in the opposite petaloid appendage (Tian *et al.* 2018). With the significant expression changes of the adaxial–abaxial polarity genes, the asymmetry of the stamen becomes more and more evident.

### Adaxial–abaxial polarity-related stamen morphogenesis in *C. indica*

The adaxial–abaxial polarity in the *C. indica* stamen that we observed has some similarities with other species. In the *rol* mutant of rice, the half-abaxialized stamen primordium develops to an asymmetric one-theca stamen (Toriba *et al.* 2010), to which the one-theca *C. indica* stamen shows a similar pattern in morphology and adaxial–abaxial polarity (Tian *et al.* 2018). In arabidopsis and rice, when the two thecae primordium emerge, the adaxial polarity shifts from the adaxial side to the two lateral sides of the stamen primordium (Toriba *et al.* 2010; Li *et al.* 2019). At the corresponding stages in *C. indica*, *CiPHB1* shows similar expression patterns to those in arabidopsis and rice (Fig. 6B). Proper expression of *AtPHB* between the inner and outer microsporangia is essential for the separation of the two microsporangia and the formation of the stomium regions in arabidopsis (Li *et al.* 2019). The similar expression pattern of *CiPHB1* in the one-theca anther of *C. indica* indicates that *CiPHB1* may undertake the same function (Fig. 6B).

Recently, a model was proposed that accounts for the formation of cup-shaped leaf traps of *Utricularia gibba* (Lentibulariaceae) through shifts in adaxial–abaxial gene expression (Whitewoods *et al.* 2020). In this model, growth is oriented by adaxial–abaxial domains acting throughout the leaf rather than just at the epidermal boundary. Accordingly, besides the force from outer whorl organs, the inward curling of the petaloid appendage which ‘traps’ the style, may also be positively regulated by the expression of adaxial–abaxial polarity genes in the middle part of the petaloid appendage (Fig. 6B).

To summarize, we revealed the irregular dynamic expression patterns of adaxial–abaxial polarity marker genes during the development process of the *C. indica* stamen. In general, the expression patterns of markers changed simultaneously with, if not earlier than, the corresponding morphological changes in the stamen, raising the possibility that *CiPHB1* and *CiFIL* regulate stamen morphogenesis in *C. indica*.

## Supporting Information

The following additional information is available in the online version of this article—

**Table S1**. Gene information and primer pairs used to generate hybridization probes.

plaa051_suppl_Supplementary_Table_S1

## Notes

**Form & Function. Chief Editor**: Kate McCulloh
