# Supplementary material for: Irregular adaxial–abaxial polarity rearrangement contributes to the monosymmetric-to-asymmetric transformation of Canna indica stamen
Source: AoB Plants. 2020 Sep 11;12(5):plaa051. doi: 10.1093/aobpla/plaa051 (PMC7590949; doi:10.1093/aobpla/plaa051)
Supplement: plaa051_suppl_Supplementary_Table_S1 [file plaa051_suppl_supplementary_table_s1.pdf]

Supporting Information—Table S1. Gene information and primer pairs used to generate hybridization probes.

Gene name	Gene_ID*	Direction	Primer sequence
CiPHB1	CL1221.Contig2_All	Forward	AGAATGTGCCACCTGCGATT
		Reverse	TCAAACAAAAGACCAGTTCAC
CiFIL	CL8364.Contig2_All	Forward	TCTCTTCAAGACCGTGACCGT
		Reverse	TTTCGTCCTTGATGAAGCGA
AtPHB	AT2G34710	Forward	GAATCCGCCTGCGGCCTCGTGAGTT
		Reverse	GGATCCTTCTAAATCGGAGGCT
AtFIL	AT2G45190	Forward	ACCGTAACTGTCCGATGT
		Reverse	GAGCCCGAAGTGTATGTG

* The unigenes of *Canna indica* were generated by transcriptome Illumina sequencing. The data was deposited in NCBI Gene Expression Omnibus (GEO) (Accession GSE72440). Sequence data of *Arabidopsis thaliana* from this article can be found in the GenBank database under the accession numbers (Gene ID).
